# The impact of widowhood on the mental health of older adults and the buffering effect of social capital

**DOI:** 10.3389/fpubh.2024.1385592

**Published:** 2024-04-24

**Authors:** Jichao Zheng, Lei Yan

**Affiliations:** ^1^Department of Economics Research, Anhui Academy of Social Science, Hefei, Anhui, China; ^2^School of Marxism, Jiangsu University of Science and Technology, Zhenjiang, Jiangsu, China

**Keywords:** widowhood, aged, mental health, social capital, buffering effect, community-level social capital, family-level social capital

## Abstract

**Background:**

Widowhood is one of the most serious issues affecting the mental health of older persons. China currently has tens of millions of widowed older adult, which is a huge group. It is of great significance to study the impacts of widowhood on their mental health and put forward some measures for improvement.

**Method:**

We used China Family Panel Studies (CFPS) data in 2020, which included 4,184 older adults. Linear regression is used to examine the relationship among widowhood, mental health, and social capital.

**Results:**

Both short-term and medium- and long-term widowhood lead to a significant increase in depression, which seriously affects the mental health of older people. At the same time, community-level and family-level social capital have significant buffering effects on the loss of mental health caused by widowhood, but this effect is heterogeneous, with different types of social capital playing different roles among different gender groups.

**Conclusion:**

The provision of care support by children and good neighborhood relationships can help mitigate the psychological impact of widowhood, and these are areas where social policy can make a difference.

## 1 Introduction

Mental health problems can significantly impact older persons' ability to carry out basic activities of daily living (1). By 2022, the number of people over 65 in China has exceeded 200 million, accounting for 14.9% of the total population (2). This means that China has entered an aging society. So it is more and more important for older people to spend their twilight years happily to achieve healthy aging. Aging, the increase in average life expectancy, and the difference between male and female life expectancy have led to a steady rise in the number of widowed older persons. According to data from the 10% sample of the 2020 Census, there are 5,566,000 widowed people over 60, of which 1,496,000 are men and 4,070,000 are women (3). Using the above data as a basis for estimation, the number of widowed older adult could be tens of millions. This is a huge group of people, so it is of great significance to study the impact of widowhood on their mental health and to propose measures and paths for improvement.

Research has shown that widowhood has a severe negative impact on the mental health of older people. Some studies have identified widowhood as the most prominent life crisis facing more senior people, ranking first on the Geriatric Social Readjustment Rating Scale (GSRRS) (4). Widowhood can hurt older people's mental health; for example, the highest risk factor for depression is widowhood (5). Older widows often experience loneliness, depression, and anxiety (6). Widowhood can also lead to a decrease in psychological wellbeing and an increase in the proportion of older people who feel unhappy (7).

Numerous studies have shown that social capital is closely related to the mental health of older adults, and that social capital significantly enhances the mental health of older adults. Social capital provides individuals with a sense of acceptance and belonging, thus providing a sense of security that their needs will be met, contributing to their physical and mental wellbeing (8). Social capital is believed to help buffer mental stress, help people cope with it and maintain positive emotions in their daily lives (9). In general, social capital such as social trust and social networks have a significant positive impact on an individual's mental health (10).

Note that widowhood and social capital have opposite effects on older people's mental health. Some scholars began to pay attention to the role of social capital in supporting the mental health of widowed older adults. Some studies have suggested that social capital plays a vital role in the recovery of the mental health of widowed older adults and that social support is an essential moderator of older adults' adaptation to bereavement (11). For example, social capital is particularly important in the recovery of traumatic grief (12). However, not much literature examines the mitigating role of social capital, empirical evidence based on Chinese data is even rarer. In a sense, the buffering role of social capital is still a theoretical gap, and given that there are so many widowed older people in China, clarifying the buffering role of social capital can help to better develop policy initiatives to enhance their mental health.

The primary aim of our study was to identify whether widowed older adults with more social capital have better mental health. A second objective was to examine the gender differences in the buffering effect of social capital. Finally, we aim to use the findings to provide evidence-based recommendations to alleviate depression among widowed older persons.

## 2 Literature review

### 2.1 Social capital and mental health

Social capital refers to the characteristics of social organization, including trust, norms, and networks, and social capital can improve social efficiency by facilitating coordinated action (13). Social capital can be categorized into cognitive social capital, which includes cognitive-level social capital, such as trust and norms, and structural social capital, such as observable social connections and activities (14).

In general, the role of social capital on specific physical health is not apparent, and few studies have found significant associations between social capital and obesity, diabetes, or infectious diseases. However, social capital has stronger effects on psychological and mental health (15). For example, increases in average social trust at the national level lead to improved self-rated health (16). Older adults with lower levels of interpersonal trust or reciprocity are more likely to experience depressive symptoms than those with higher levels of interpersonal trust or reciprocity (17). People with low levels of social trust in general are more than three times as likely to experience depression, three times as likely to experience many psychosomatic symptoms, and twice as likely to experience many musculoskeletal pain symptoms (18).

### 2.2 The impact of widowhood on the mental health of older persons

Widowhood is a significant change in the lives of older people and can have a profound impact on their mental health. Widowhood significantly reduces older adults' social capital and affects their mental health through several pathways. First, marriage is an important family-level social capital. Widowhood deprives older people of the protective effects of marriage. Some scholars believe that the impact of widowhood on older people's mental health can be understood as the long-term difference between the beneficial attributes of marriage and the harmful attributes of widowed life (19). The protective effect of marriage is that it brings companionship, and companionship reduces loneliness, and older adults who have lost their partners are lonelier (20). Moreover, spouses provide emotional comfort in the face of various internal and external stressors, such as care in times of illness, encouragement to adopt healthful lifestyles (21), and attention to and encouragement of healthy behaviors by spouses (22). In contrast, widowed individuals are more likely than married individuals to engage in unhealthy behaviors such as drinking and driving, substance abuse (23), and more “risk-taking behaviors” such as driving too fast, and getting into serious arguments or fights (24).

Second, because the spouse is the most important social relationship, widowhood deprives older persons of their most important social capital, thus undermining the social support available to them (25). For example, recently widowed men may find it challenging to maintain their social networks in the absence of their wives (26); similarly, for widows, the loss of their late husband is an important way of connecting them to society, and widowhood cuts off this social link (27). In short, widowhood causes the loss of lasting emotional bonds formed with others over a long period, and the loss of significant family social capital, which triggers psychological processes of grief and mourning (28).

### 2.3 Analysis of the interactions between widowhood, social capital, and mental health

As mentioned above, widowhood can lead to a lack of marriage-related social capital, which can affect an individual's mental health. However, social capital is diversified and multi-level; can other aspects of social capital compensate for the damage to mental health caused by widowhood? The social capital buffer theory has the potential to provide answers. The buffering effect theory states that individuals can use social capital as a buffer against the adverse effects of various stressful events on their health (29). Social capital can affect mental health through two mechanisms; one is that social capital can directly attenuate an individual's evaluative response to a stressful event (30). The second mechanism is that perceived differences in social capital can impact mental health, and the belief that others will provide necessary resources can dampen emotional and physiological responses to stressful events or alter maladaptive behavioral responses (31).

According to the buffering effect theory, other types of social capital may play a buffering role in the mental health loss caused by widowhood. For example, research has found that the more positive support children give to the bereaved person in the 6 months following bereavement, the fewer depressive symptoms in the bereaved (32). In the early stages of grief, adult children of the bereaved meet their needs by reducing negative social interactions (33). Surprisingly, a search of the existing literature reveals that few studies have focused on the moderating effects of social capital on widowhood, and these studies have had conflicting results; for example, one study found that increases in structural social capital may attenuate the effects of spousal bereavement on depressive symptoms, yet these associations varied by gender, living arrangement, and duration of grief (34). But other studies have found that the social capital may not be sufficient to protect bereaved spouses from deteriorating mental health (35), and there was no evidence of a buffering or a recovery effect of social capital (36).

As far as the existing research is concerned, social capital will have an impact on people's mental health in various ways, and in general, more social capital means better mental health; in addition, widowhood, as a major blow in the course of one's life, will have an impact on the mental health of the person concerned in the short term, but this impact will gradually dissipate in the long term. Whether social capital can play a buffering role in the mental health damage induced by widowhood is not clear. Whether this moderating effect is related to gender requires further research. This study will make a marginal contribution in this area based on the latest CFPS data. Specifically, we will focus on the different effects of short-term widowhood and medium- and long-term widowhood status on the mental health of older adults and examine whether the buffering effect of social capital between widowhood and older adults' mental health exists, and whether this moderating role is heterogeneous across groups.

## 3 Methodology and design

### 3.1 Data sources

This study used the China Family Panel Studies (CFPS) 2020 and 2018 summary questionnaires. The survey aims to reflect changes in China's society, economy, demographics and perceptions by tracking and collecting data at the individual, household and community levels, providing a database for academic research and public policy analysis. The China Family Panel Studies (CFPS) is an ongoing, and longitudinal social survey covering most of the country. The CFPS is conducted by Peking University and covered 25 of the 31 provincial units in the mainland of China. The baseline CFPS survey in 2010 successfully interviewed 14,960 households and 42,590 individuals living in those households (37). The CFPS 2018 survey completed ~15,000 household interviews and collected about 44,000 individual questionnaires (38). The CFPS 2020 covered more than 22,000 household units, taking the number of households completed in 2018 as the denominator; the cross-round response rate at the household level was 77% (39). The current retirement age in China is 60, and in Chinese society, people over the age of 60 are generally considered to be older adult. In line with the purpose of the study, we limited the study samples to older adults over the age of 60, who had no missing values for any of the variables we selected.

### 3.2 Dependent variables

Depression is the most common cause of emotional distress in later life of older adults (40). We chose depression values to represent mental health. The CFPS uses the 8-item Center for Epidemiologic Studies Depression Scale to measure depressive symptoms. The 8-item scale is concise, which reduces respondent burden and ensures data quality (41). Some studies have shown that the psychometric results of the 8-item scale are comparable to those of the full scale (42). And the 8-item scale is a valid and reliable measure of depressive symptoms in community-dwelling older adults compared to the full scale (43). The 8-item scale consists of six positively scored questions, such as “I am bothered by small things.” and two negatively scored questions, such as “I am hopeful about the future.” Each question had four answers: rarely or not at all, not too much, sometimes or half the time, and most of the time; each answer was coded 1, 2, 3, or 4 for the positively scored questions, and 4, 3, 2, or 1 for the negatively scored questions. We summarized the answers to the eight questions and constructed a continuous variable ranging from 0 to 32, with higher values indicating more severe depression.

### 3.3 Explanatory variables

As noted in the literature review, short-term and long-term widowhood have different effects on mental health. Therefore, when setting explanatory variables, the explanatory variables include short-term widowhood, medium- and long-term widowhood, and social capital. The short-term widowhood stems from the change in respondents' marriages between 2018 and 2020; those respondents who were in marriages in 2018 and whose marital status changed to widowed in 2020 can be considered to have experienced a short-term widowhood shock during these 2 years; while medium- and long-term widowhood refers to respondents whose marital status are widowed in both 2018 and 2020.

For the social capital variables, we used cognitive social capital at the neighborhood level and structural social capital at the household level, respectively. Neighborhood-level social capital has a strong impact on mental health, and prior research has shown that the more trust respondents have in other community members, the better their mental health; the more community members help each other, the less time members spend experiencing despair, feelings of failure, loneliness and meaninglessness (44). Trust in neighbors and perceptions of neighborhood cohesion reduced the likelihood of depressive symptoms (45). Therefore, for community-level perceived social capital, we used the neighborhood relationships variable and the anticipated help from neighbors variable, both of which were answered by a representative of the respondents in the household, and we used the options they answered to represent their family members' perceptions of neighborhood cohesion and trust.

For the neighborhood relationships variable, the questionnaire asked, “How do you feel about the neighborhood in the (village/sub-district) where you live?” with the following options: 1. very good, 2. good, 3. fair, 4. poor, and 5. very poor, we assign values from 5 to 1 to each response, respectively. For the anticipated help from neighbors variable, the questionnaire asked, “Do you think that someone will help you if you need help from your neighbors?” with the following options: 1. Certain, 2. Maybe, 3. Not sure, 4. Not very likely, 5. Impossible, we assign values from 5 to 1 to each response, respectively. The family-level social capital is the child care variable, where the questionnaire asked, “In the past 6 months, have your children taken care of your household chores or meals? How often did you receive care?” the answers to this question are: 1. Almost every day, 2. 3–4 days a week, 3. 1–2 days a week, 4. 2–3 days a month, 5. 1 day a month, and 6. 1 day a few months. We assigned a value of 1 to “almost every day” and “3–4 days a week” and a value of 0 to the other cases.

### 3.4 Control variables

Like many existing studies, the control variables mainly include demographic background factors such as gender and type of residence, individual socio-economic status such as personal education and decile ranking of per capita household income in the province where the individual lives (46), and health status. Table 1 presents the original questions and codes for all control variables.

**Table 1:** Summary of the control variables.

**Variable**	**Original question**	**Code**
Age	What's your birth data?	Year of visit minus year of birth.
Gender	Respondent's gender.	Male = 1, Female = 0.
Type of household	What is your current household status?	Rural household = 1, others = 0.
Education	The highest level of education?	The respondents were categorized into three groups: low education (elementary school and below), medium education (middle school), and high education (high school and above).
Work	Did you work at least 1 h in the past week?	A “yes” answer is assigned a value of 1, meaning the respondent is working.
Family income ranking	What is the per capita household income?	Per capita household income is ranked by decile according to the province in which the household is located, with scores ranging from 1 to 10; the higher the score, the higher the household income.
Physical functional limitations	We will mention a variety of activities and ask you to judge whether you can do them independently.	Independence in outdoor activities, independence in eating, independence in kitchen activities, independence in public transportation, independence in shopping, independence in cleaning, and independence in laundry, a total of seven activities, and answering “no” to any of them assigns a value of 1. The variable takes on a value range of 0–7.
Hospitalization	Have you been hospitalized for a medical condition in the past 12 months?	The answer “Yes” is assigned a value of 1 and “No” is assigned a value of 0.
Illness	In the past 6 months, has a doctor diagnosed you with a chronic disease?	The answer “Yes” is assigned a value of 1 and “No” is assigned a value of 0.
Drinking	In the past month, did you drink alcohol more than 3 times a week?	The answer “Yes” is assigned a value of 1 and “No” is assigned a value of 0.

### 3.5 Research methodology

We used multiple regression methods to examine the effects of short-term widowhood shocks and medium- and long-term widowhood on the mental health of older adults. An interaction term between widowhood and social capital is also added to the multiple regression equation to test the buffering effect of social capital on widowhood by examining the coefficient of the interaction term:


(1) $$\begin{array}{lll} Depression_{i} & = & a+\beta_{1}Widowhood_{i}+\beta_{2}SocialCapital_{i}\\ \;+\beta_{3}Widowhood_{i}*SocialCapital_{i}\\ \;+\beta_{n}ControlVariables_{in}+\varepsilon_{i} \end{array}$$

In Equation (1), depression_i_ value indicates the mental health of the respondent_i_. Widowhood_i_ is the dummy variable indicating whether or not the respondent is widowed. SocialCapital_i_ is the personal social capital variable, Widowhood${\;}_{\text{i}}^{*}$SocialCapital_i_ is the interaction term, and ControlVariables_i_ are the control variables.

From Equation (1), it is easy to see that the marginal effect of the widowhood variable on mental health is:


(2) $$\begin{array}{l} \frac{\partial Depression}{\partial Widowhood}=\beta_{1}+\beta_{3}SocialCapital \end{array}$$

In Equation (2), β1 represents the direct effect of widowhood on mental health, and according to many existing studies, the coefficient of this variable is usually positive; β3 represents the coefficient of the interaction term, and if the coefficient of β3 is negative, it means that social capital has a buffering effect on mental health shocks caused by widowhood, and that social capital can play a moderating role between widowhood and mental health.

## 4 Empirical results

### 4.1 Data description

The descriptive statistics for each variable are described in Table 2. There are 4,184 respondents in the sample who were married or widowed in 2018 and 2020, of which 3,691 were married, and 493 were widowed in 2018. It is easy to see that in the same year, the depression values of the widowed persons are significantly higher than those of the married persons. Moreover, widowed persons experienced a significant increase in depression values between 2018 and 2020, which intuitively shows that short-term widowhood has a greater psychological impact. Meanwhile, the depression values of medium- and long-term widowed persons decreased, which suggests a gradual psychological adaptation of individuals to the event of widowhood over time. There was a substantial increase in the proportion of receiving care from children for both short-term and medium- and long-term widowhood, showing that children provide essential support in terms of caregiving resources in the absence of spousal care. Among the control variables, the most significant difference between widowed and married persons is a decrease in the proportion of widowed persons participating in the workforce, i.e., people work less after being widowed, as well as the significant increase in the likelihood of hospitalization among widowed persons; furthermore, it should not be overlooked that the average age of widowed persons is higher than that of married persons.

**Table 2:** Descriptive statistics of the variables.

**Variable**	**Male**	**Female**	**Short-term widowhood**	**Medium- and long-term widowhood**	**Married**
Married	2,012	1,583			3,595
Short-term widowhood	31	65	96		
Medium- and long-term widowhood	145	348		493	
Depression-2020	12.80256 (4.19772)	14.27455 (4.61672)	15.20833 (5.26341)	14.83367 (4.61844)	13.27705 (4.37712)
Depression-2018	12.71344 (3.92810)	14.23447 (4.50387)	14.11458 (4.507)	15.20284 (4.95051)	13.17914 (4.11374)
Neighborhood relationships	4.104205 (0.83593)	4.14429 (0.84854)	4.07292 (0.83659)	4.121704 (0.82725)	4.124896 (0.84446)
Anticipated help from neighbors	4.547075 (0.80640)	4.52054 (0.85786)	4.59375 (0.78911)	4.427992 (0.95710)	4.547427 (0.81285)
Receive child care	0.175046 (0.38009)	0.21944 (0.41397)	0.38542 (0.48925)	0.3204868 (0.46714)	0.1741307 (0.37927)
Age	68.14397 (5.84496)	68.05311 (5.63752)	70.75 (6.43347)	72.06694 (6.86999)	67.48595 (5.30610)
Type of household	0.67322 (0.46914)	0.68337 (0.46528)	0.70833 (0.45692)	0.69777 (0.45969)	0.674548 (0.46861)
Education-low	0.5169104 (0.49983)	0.72646 (0.44589)	0.71875 (0.45197)	0.7707911 (0.42075)	0.59305 (0.49133)
Education-medium	0.2755941 (0.44692)	0.16283 (0.36930)	0.1875 (0.39236)	0.131846 (0.33867)	0.23505 (0.42409)
Education-high	0.2074954 (0.40561)	0.1107214 (0.3138655)	0.09375 (0.29301)	0.0973631 (0.29675)	0.17191 (0.37735)
Family income ranking	4.622486 (2.76616)	4.588176 (2.806677)	4.45833 (2.89797)	4.537525 (2.79247)	4.61947 (2.78178)
Physical functional limitations	0.33227 (1.08728)	0.4579158 (1.141925)	0.4375 (1.04441)	0.6186613 (1.38686)	0.35994 (1.07123)
Hospitalization	0.17230 (0.37773)	0.1968938 (0.397751)	0.23958 (0.42907)	0.212982 (0.40983)	0.17858 (0.38305)
Work	0.58821 (0.49227)	0.4539078 (0.4979957)	0.32292 (0.47005)	0.3509128 (0.47774)	0.553277 (0.49722)
Drinking	0.2769653 (0.44760)	0.0370741 (0.18899)	0.1875 (0.39236)	0.1034483 (0.30485)	0.1699583 (0.37565)
Illness	0.27468 (0.446455)	0.3261523 (0.4689213)	0.3229167 (0.4700457)	0.3387424 (0.4737629)	0.293185 (0.4552859)
*N*	2,188	1,996	96	493	3,595

### 4.2 Interaction statistical plots of social capital, depression value, and marital status

**Figure 1:**
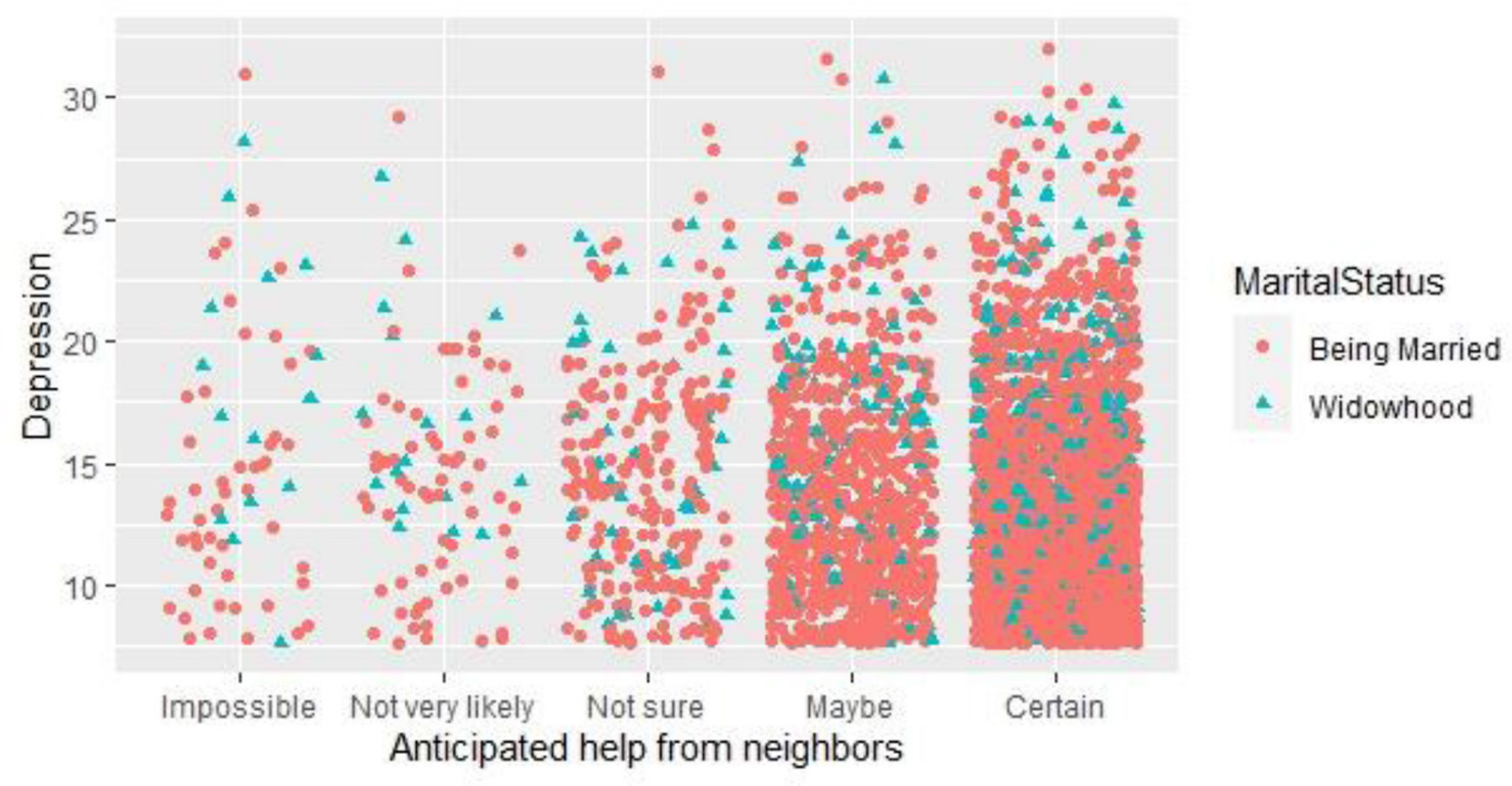
Interaction statistics between anticipated help from neighbors and depression value.

The interactions of social capital, depression value, and depression value in 2020 are shown in the following three figures. Figure 1 shows the interaction statistics of anticipated help from neighbors, depression value, and marital status. In terms of the anticipated help from neighbors, most people think that they will have a neighbor to help them. At the same time, from the frequency of distribution of widowed and married people in each option, the depression value of widowed people is higher than that of married people, which indicates that the distribution of widowed people in the domains of higher depression value is higher.

**Figure 2:**
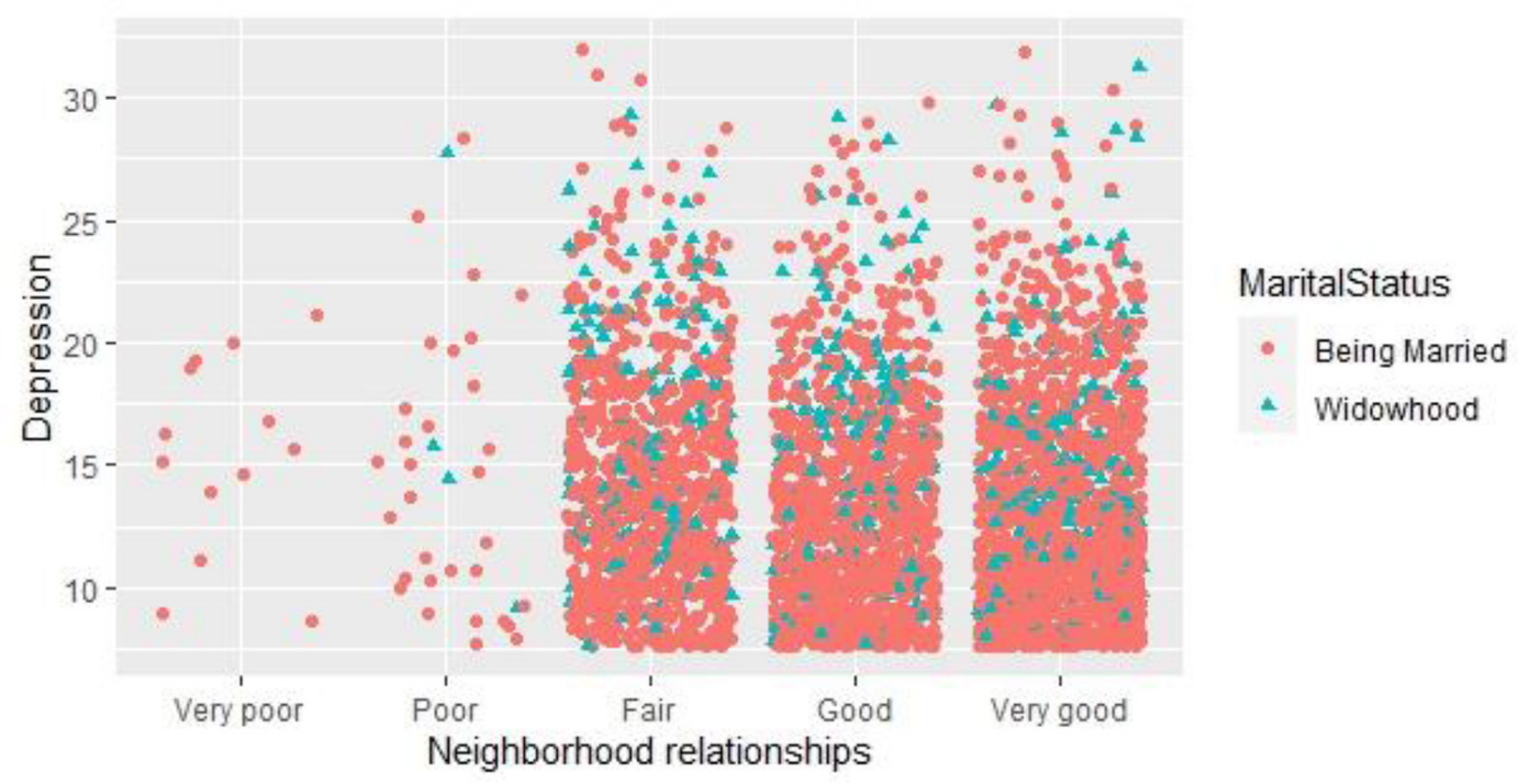
Interaction statistics between neighborhood relationships and depression values.

Figure 2 shows the interaction statistics of neighborhood relationships with depression value and marital status. In term of the neighborhood relationships, fewer people chose “very poor” and “poor” neighborhood relationships, and most people chose “good” and “very good” neighborhood relationships. Among those who chose “very poor” and “poor” neighborhood relationships, fewer people were widowed.

**Figure 3:**
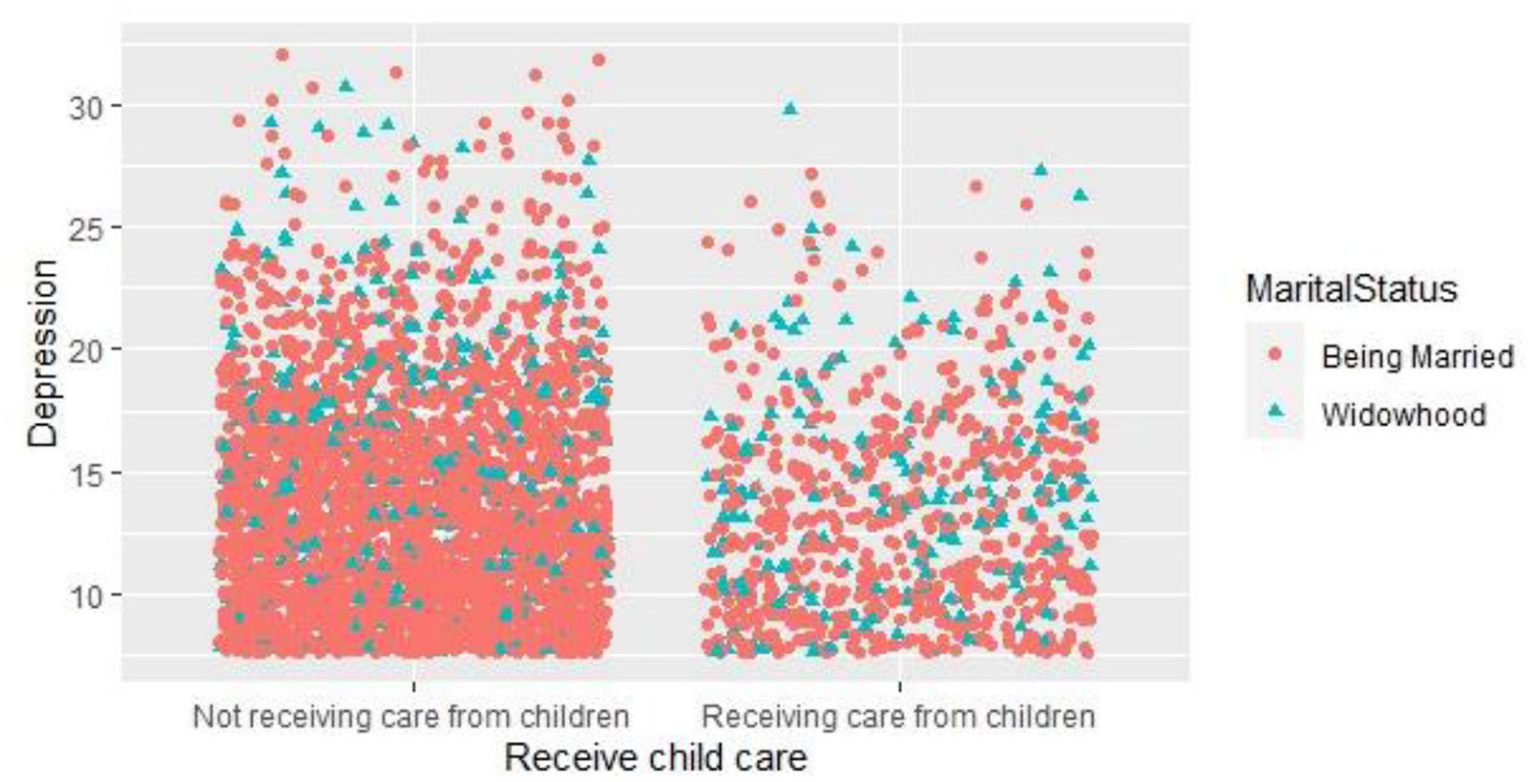
Interaction statistics between receive child care and depression values.

On the interaction statistics of child care, depression value, and marital status, we can see from Figure 3 that the majority of respondents did not receive child care, but a small proportion of respondents did; it can also be visualized from the interaction plot that the proportion of widowed respondents who accepted child care was significantly higher than the proportion of widowed respondents who did not receive child care, indicating that in the absence of spousal support, children will pay more attention to and take care of their widowed parents.

### 4.3 Overall sample regression

We use three multiple regression equations for the overall sample regressions and the results are presented in Table 3. Model 1 contains only short-term widowhood, medium- and long-term widowhood, and control variables, while Models 2 and 3 include interaction terms between social capital and the widowhood variable to analyze the effect of social capital on widowhood. Model 2 includes the interaction terms between the short-term widowhood and the social capital variables. Model 3 includes the interaction terms between the medium- and long-term widowhood and the social capital variables to observe the buffering effect of social capital on widowhood.

**Table 3:** Multiple regression in total sample.

**Variables**	**Model 1**	**Model 2**	**Model 3**
Short-term widowhood	1.40521^***^ (0.38978)	1.48187 (2.72902)	1.37734^***^ (0.38981)
Medium- and long-term widowhood	0.43016^**^ (0.18693)	0.43104^**^ (0.18700)	2.23900^**^ (1.07625)
Depression-2018	0.43754^***^ (0.01457)	0.43748^***^ (0.01458)	0.43650^***^ (0.01458)
Gender	−0.65722^***^ (0.12888)	−0.65860^***^ (0.12892)	−0.66173^***^ (0.12885)
Type of household	0.41521^***^ (0.15431)	0.41205^***^ (0.15439)	0.39757^**^ (0.15442)
Education-medium (Ref: Education-low)	−0.16045 (0.15094)	−0.15959 (0.15100)	−0.16449 (0.15089)
Education-high (Ref: Education-low)	−0.14275 (0.17902)	−0.14196 (0.17906)	−0.14694 (0.17895)
Family income ranking	−0.09783^***^ (0.02346)	−0.09855^***^ (0.02348)	−0.09706^***^ (0.02345)
Physical functional limitations	0.27148^***^ (0.05387)	0.27144^***^ (0.05389)	0.27267^***^ (0.05388)
Hospitalization	0.97287^***^ (0.15771)	0.97627^***^ (0.15784)	0.97235^***^ (0.15767)
Work	0.41319^**^ (0.13301)	0.41514^**^ (0.13307)	0.41471^***^ (0.13299)
Drinking	−0.06240 (0.16634)	−0.06449 (0.16642)	−0.06617 (0.16631)
Illness	0.82601^***^ (0.13299)	0.82124^***^ (0.13308)	0.83007^***^ (0.13299)
Receive child care	−0.45671^***^(0.14779)	−0.46817^***^ (0.15039)	−0.31641^*^ (0.16147)
Anticipated help from neighbors	−0.21728^***^ (0.07407)	−0.22668^***^ (0.07495)	−0.20598^**^ (0.08027)
Neighborhood relationships	−0.20164^***^ (0.07288)	−0.19026^***^ (0.07380)	−0.16400^**^ (0.07721)
Receive child care^*^ Short-term widowhood		0.41538 (0.81296)	
Anticipated help from neighbors^*^ Short-term widowhood		0.36409 (0.50115)	
Neighborhood relationships^*^ Short-term widowhood		−0.46787 (0.47947)	
Receive child care^*^ Medium- and long-term widowhood			−0.82627^**^ (0.39688)
Anticipated help from neighbors^*^ Medium- and long-term widowhood			−0.02485 (0.20561)
Neighborhood relationships^*^ Medium- and long-term widowhood			−0.35248 (0.23182)
Adjusted *R*^2^	0.3009	0.3006	0.3016

^*^P < 0.1; ^**^P < 0.05; ^***^P < 0.01.

The regression results of model 1 show that short-term widowhood has a powerful psychological impact, with widowhood within the last 2 years leading to an average increase of 1.43 in depression values. And medium- and long-term widowhood also leads to an increase of 0.47 in depression value. So, it is clear that short-term widowhood has a much larger impact on mental health. The three variables representing social capital “receiving child care,” “neighborhood relationships,” and “anticipated help from neighbors” all have negative and significant effects on depression, indicating that the higher the social capital, the lower the depression value. Among the control variables, the 2018 depression variable significantly affected the depression value in 2020. In line with many studies, variables such as living in rural areas, physical functional limitations, hospitalization in 1 year, working, and illness are positively correlated with the depression value, whereas the variables of being male and family income ranking make the depression value decrease.

After adding the interaction terms, the regression results of Model 2 show that the interaction terms between short-term widowhood and the social capitals are insignificant, indicating that social capital has no buffering effect on short-term widowhood. The regression results of Model 3 show that among the interaction terms between medium- and long-term widowhood and the three social capitals, the coefficients of the interaction terms between receiving child care and medium- and long-term widowhood are negative and significant at the 5% level, indicating that receiving child care helps to buffer the adverse effects of medium- and long-term widowhood on mental health. Meanwhile, the interaction terms of neighborhood relationships and anticipated help from neighbors are not significant.

### 4.4 Multiple regression by gender

There are significant differences between men and women in the amount and structure of social capital they possess (47). In addition, there are differences between men and women in mental health, such as depression and anxiety levels are higher in women than in men (48). Women are more vulnerable to stressors than men, and women psychologically benefit more from social support relationships (49). And the mental health benefits of social capital vary by gender (50). Because of these gender differences, social capital may buffer the mental health of men and women through different mechanisms, so that we will conduct multiple regression by gender here. When designing the regression model, we no longer pay attention to the buffer effect of social capital on short-term widowhood shock because the overall sample regression results show that social capital has no buffer effect on short-term widowhood shock, and the number of new widowhood within 2 years is negligible. Therefore, we no longer analyze the buffer effect of social capital on short-term widowhood in the subsequent sex-grouped regression, but focus on the buffer effect of social capital on medium- and long-term widowhood.

The explanatory variables here are depression value too; the core explanatory variables are still short-term widowhood shock, medium- and long-term widowhood, and three social capital variables, and the control variables are the same as the overall sample regression. We constructed four multiple regression equations, model 1 and model 2 are regressions with male samples, model 3 and model 4 are regressions with female samples. Model 2 and model 4 also included the interaction term of social capital and medium- and long-term widowhood.

The regression results in Table 4 showed that, in the absence of interaction terms, both short-term widowhood or medium- and long-term widowhood had a higher impact on the mental health of males than females. While for females, the psychological effects of medium- and long-term widowhood are not significant. In addition, the impact of short-term widowhood was also greater than that of medium- and long-term widowhood, indicating that the mental health damage caused by widowhood would gradually flatten over time. After adding the interaction between social capital and medium- and long-term widowhood, for men, the variables “receiving child care” and “anticipated help from neighbors” can buffer the adverse effects of medium- and long-term widowhood on mental health, while the influence of neighborhood relationships variable is not clear and significant. For women, variables such as “receiving child care” and “anticipated help from neighbors” do not buffer the adverse effects of medium- and long-term widowhood, but the neighborhood relationships variables at the community level significantly buffered the effects of medium- and long-term widowhood on women's mental health.

**Table 4:** Multiple regression in male and female samples.

**Variables**	**Model 1**	**Model 2**	**Model 3**	**Model 4**
Short-term widowhood	2.13164^***^ (0.65167)	2.10020^***^ (0.65110)	0.98956^**^ (0.49824)	0.97149^*^ (0.49855)
Medium- and long-term widowhood	0.82843^***^ (0.31688)	3.38626^*^ (1.81962)	0.24768 (0.23791)	1.93840 (1.40165)
Receive child care	−0.32420 (0.20276)	−0.19838 (0.21231)	−0.57751^***^ (0.21608)	−0.46293^*^ (0.24662)
Anticipated help from neighbors	−0.31701^***^ (0.10096)	−0.24759^**^ (0.10623)	−0.12034 (0.10923)	−0.15656 (0.12183)
Neighborhood relationships	−0.25783^***^ (0.09762)	−0.27038^***^ (0.10046)	−0.13979 (0.10923)	−0.03859 (0.11935)
Receive child care^*^ Medium- and long-term widowhood		−1.20208^*^ (0.70470)		−0.46021 (0.50854)
Anticipated help from neighbors^*^ Medium- and long-term widowhood		−0.61973^*^ (0.33963)		0.22865 (0.26868)
Neighborhood relationships^*^ Medium- and long-term widowhood		0.11876 (0.40957)		−0.61904^**^ (0.29531)
Control variables	Yes	Yes	Yes	Yes
*N*	2,188	2,188	1,996	1,996
Adjusted *R*^2^	0.2775	0.279	0.2861	0.287

^*^P < 0.1; ^**^P < 0.05; ^***^P < 0.01.

## 5 Discussion and recommendations

Social capital is the goodwill shown to us by others, and it is a resource that people can utilize in their social relationships (51). Our research has shown that short-term widowhood is associated with significant psychological stress and that social capital is insufficient to buffer this stress. But for those who have been widowed for a more extended period, social capital, such as good neighborhood relations and caring support from children, helps to compensate for the negative effects of spousal deprivation and has a very significant protective effect on mental health, partially buffering the adverse effects of widowhood.

Firstly, widowhood over the past 2 years has had a negative impact on mental health, leading to an increase in depression scores. In addition, over the medium to long term, widowhood continues to have a lasting effect on older persons' mental health, although to a much reduced extent compared to short-term widowhood. This is consistent with many previous studies, which suggest that because short-term widowhood experiences a variety of negative emotions-deep sadness, anxiety, anger, the health risks of widowhood are highest between the 1st year and 18 months after widowhood (52). Compared to married people, newly widowed people have nearly nine times the overall depression rate and nearly four times the incidence of depressive symptoms, respectively (53), and in the medium to long term, most people absorb this short-term shock effect, such as after about 3 years, most widowed women's mental health improved (54).

Secondly, social capital does not buffer the psychological impact of short-term widowhood. This finding is consistent with existing study. Just as one study points out, in the early stages of a loved one's death, grieving people tend to be unresponsive to relationships and may even become hostile to well-intentioned help (55). Some scholars have even suggested that there is a positive correlation between distress and social support, as those widows who are most distressed may garner more social support than those who are less distressed (56). Short-term widowhood makes people's lives change dramatically in a short time, which has a great impact on mental health. In a short time after losing a spouse, people fall into grief and need a long period of adjustment to adjust their lives, so they cannot quickly obtain buffer effects from social capital. The empirical results show that short-term widowhood will produce a greater psychological impact and bring sadness. Under this emotion, although the existence of social capital can comfort the psychology of the older adult; it is not enough to buffer the impact of widowhood.

Thirdly, social capital plays a buffer role in the impact of medium- and long-term widowhood on the mental health of older people. This is not consistent with some of the existing studies, a review of the literature suggests that social support has an effect on depressive symptoms, but there is no indication of a buffering effect (36). A Japanese study concluded that social capital does not buffer people who have been widowed for more than 2 years (34). The reason for the difference may be due to differences in China's national conditions, such as the fact that Chinese people attach particular importance to community neighborhoods, in rural area, the majority of farmers' social interactions occurred in neighborhoods; in cities, densely populated apartment complexes also increase the frequency of neighborhood interactions (57). At the same time, traditional filial piety also makes older people have high expectations of their children's support (58). However, there is heterogeneity in this buffering effect, and different types of social capital have different buffering effects for respondents of different genders. As we often observe, men tend to be poor at managing family affairs and are vulnerable to life after widowhood; the variable of receiving child care and anticipated help from neighbors can help ease their psychological pain. For women, however, it is generally not difficult to manage family affairs; women are better at verbal communication, and good neighborhood relationships help mitigate the adverse effects of widowhood on mental health.

Our research findings suggest that, in terms of coping with the mental health effects of widowhood, changing the state of living alone, increasing the frequency of neighborhood interactions, and building good neighborhood relationships can help to mitigate the psychological impact of widowhood. This finding is the same as an American study which showed that social support could moderate the effect of the death of a spouse on depressive symptomatology (59). Participation in activities such as bridge clubs after widowhood has been found to foster a sense of belonging and increase opportunities to develop friendships and social support relationships, thereby reducing loneliness (60). The promotion of community activities at the community level can also contribute to the psychological wellbeing of widowed older people (61). Therefore, it is recommended that more neighborhood interaction activities be provided at the community level. On the other hand, since older adults can easily fall into a helpless situation after being widowed due to the lack of spousal care, relatives and children should try to provide companionship and care so that older people can change their state of living alone. At the same time, the family and children should be more open and tolerant toward the remarriage of older people.

To the best of our knowledge, few articles examine the buffering effect of social capital on depression based on Chinese data, and this paper makes an important marginal contribution in this area. The shortcoming of this study is that although we used data from two waves of the study, we did not use the fixed-effects analysis method and only included the 2018 depression values in the regression model because the adjustment of the statistical methods of some variables made the before-and-after comparisons impossible. Therefore, this paper still adopts a cross-sectional analysis method, which cannot utilize information on the dynamics of individual changes between 2018 and 2020 to reveal causal relationships better. Meanwhile, due to the availability of data, categorizing the duration of widowhood into short-term widowhood of <2 years and medium- and long-term widowhood of more than 2 years may be too crude to more accurately determine the mitigating role of social capital, as short-term widowhood is usually defined as <1 year according to many studies. However, as far as the available data are concerned, we cannot make a more precise division.

## Data availability statement

Publicly available datasets were analyzed in this study. This data can be found at: https://www.isss.pku.edu.cn/cfps/sjzx/gksj/index.htm.

## Ethics statement

The studies involving humans were approved by the Biomedical Ethics Review Committee of Peking University (IRB00001052–14010). The studies were conducted in accordance with the local legislation and institutional requirements. Written informed consent for participation was not required from the participants or the participants' legal guardians/next of kin in accordance with the national legislation and institutional requirements.

## Author contributions

JZ: Conceptualization, Data curation, Methodology, Writing – original draft, Writing – review & editing. LY: Data curation, Writing – review & editing.
